# Knowledge and Practice about Rabies among Children Receiving Formal and Informal Education in Samaru, Zaria, Nigeria

**DOI:** 10.5539/gjhs.v4n5p132

**Published:** 2012-08-09

**Authors:** Asabe Adamu Dzikwi, Ayuba Sini Ibrahim, Jarlath Udoudo Umoh

**Affiliations:** 1 Department of Veterinary Public Health and Preventive Medicine, Ahmadu Bello University, Zaria, Nigeria

**Keywords:** children, education, formal, informal, Nigeria, rabies, Zaria

## Abstract

**Background:**

Every year, about 50,000 people die of rabies of which about 55% of the mortalities occur in Asia and over 40% in Africa. Children are victims of up to 50% of these mortalities. The figure is alarming and immediate action is required to stop this scourge.

**Aim:**

This study was carried out to assess the knowledge, attitude and practice about rabies among children attending primary schools located in the Ahmadu Bello University (ABU) premises and those outside the university as well as those receiving informal education.

**Method:**

The participants for this study were children drawn by random selection from the schools chosen by purposive sampling. With the aid of questionnaires, information was obtained from a total of 477 children with 400 from formal educational settings among 3 schools, and 77 from quaranic schools (*almajiris*) in the informal setting.

**Results:**

There was an association between parents’ occupation and type of school children were attending (p<0.05)More children receiving formal education were aware about the disease (50.8%) than those receiving informal education (32.5%), likewise those residing within ABU quarters (71%) were better informed than those residing outside ABU quarters (43.3%). Among children in the formal schools, 25.9% obtained information from friends and at school (25.9%), while in the informal setting, 56% obtained information from friends and only 16% from school. With regards to attitude and practice, 75.5% of children receiving formal education came from homes where dogs were vaccinated against rabies and and 23.3% of them play with dogs they know, while 11.1% of those receiving informal education vaccinate their dogs and fewer of them (14.3%) play with dogs known to them. There was however no association between the type of school and whether or not they play with dogs (p>0.05). Many children (65.7%) of those in formal schools know the role of dogs in rabies transmission, compared to only 8% in the informal schools. However, only 9.7% of children in formal schools associate both signs of furious and dumb form of rabies with the disease, compared with 28% in informal schools. Among children bitten by dogs, 87.5% of those receiving informal education received hospital treatment compared to 63.7% of those going to formal schools. About 13% in each of the two categories received traditional treatment. It is, therefore, important for children to be properly educated about rabies so that they can avoid dogs, recognise potential exposures, report to a responsible adult and pass on the knowledge to their peers.

## 1. Introduction

Rabies is a highly fatal disease with a case fatality rate of about 100% (Rupprecht et al., 2002). The aetiologic agent of this disease is the rabies virus belonging to the genus *Lyssavirus* and family *Rhabdoviridae* (WHO, 2005). Transmission of the disease occurs mainly following adequate contact of virus -laden saliva with broken skin, but other rare means of transmission include via aerosol, neural and infected tissues (Rupprecht et al., 2006). In many developing countries where rabies is endemic, the domestic dog (*Canis familiaris*) is the reservoir and exposure commonly occurs following a bite (Lai et al., 2005; WHO, 2005; Sudarshan et al., 2997). Clinical signs of this disease appear following migration of the virus from the bite site to the central nervous system, the duration of which is highly variable depending on several factors including the distance of the bite site to the brain (Cleaveland et al., 2002;).

Although this disease is 100% preventable with prompt and appropriate post exposure measures, it stills accounts for an estimated 50,000 human mortalities annually worldwide (WHO, 2005, www.worldrabiesday.org, 2012). Between 35% to 50% of rabies mortalities occur in children less than 15 years of age (Chhabra et al., 2004; Parviz et al., 2004; Cleaveland et al., 2002; Sudarshan et al., 2007). This information is, however, neglected in planning rabies control and prevention programmes possibly because it is relatively unknown both at the international health community and locally in rabies endemic countries (Briggs & Mahendra, 2007). Children are by nature, very curious, small in stature and often play with dogs not realising the dangers involved (Briggs & Mahendra, 2007). This frequently leads to severe bites in areas that are highly innervated and in the upper extremities of the body. The implication is that rabies transmission is faster because of the proximity of these sites to the central nervous system.

Among exposed individuals, rabies prevention is hinged on proper and prompt first aid, and post exposure vaccination. Children however do not report minor injuries either for fear of being reprimanded for playing with dogs or they just do not understand the risk involved (Briggs & Mahendra, 2007). Children learn from different sources and the type of schools they attend may provide opportunities for them to learn about the disease and its prevention. Education is a very viable tool for rabies prevention and since children are a high-risk group, there is the need to target them for this purpose.

In most of the cities in the northern part of the country, it is common to find young boys (*almajiris*) who attend quaranic schools away from their families under the authorities of their *Malams* (teachers). They are usually found begging for alms on the streets when not receiving quaranic lessons. These children generally do not receive western education and are not found in the formal conventional school setting but rather in informal school settings.

This study was therefore aimed at obtaining baseline information about the knowledge and practice about rabies among children who are receiving formal education andthose receiving informal education in Samaru, Zaria.

## 2. Study Location and Methodology

### 2.1 Study Area

The study was conducted in 2010 in Samaru district of Sabongari Local Government Area, Kaduna State. Samaru is located at 11 0 3’ N and 70 0 42’ E in the fringes of the Northern Guinea Savannah. There is an estimated 250,000 people residing in Samaru according to the 2006 Population census (National population Commision). It is a combination of residential and commercial areas, with many ethnic groups, major ones include Hausa, Fulani, Yoruba, Igbo, Ebira, Idoma etc. There is a strong presence of educational institutions in the district with primary, secondary as well as tertiary institutions located in the area.

### 2.2 Study Population and Data Collection

Following consent of the responsible authorities, children of school age (5-20), receiving formal or informal education was selected. This was achieved by purposive sampling of the schools where children who attend schools within the Ahmadu Bello University (ABU) Campus (one primary and one secondary school) and those outside ABU Campus (one secondary school), as well as those receiving informal education (ie almajiris of primary and secondary school age) formed the sample population. Children were picked following random sampling by balloting. Respondents were drawn from both sexes. A total of 477 children formed the study population.

Where children were literate, questionnaires were self-administered, otherwise, they were interviewer-administered by one person who read out the questions and translated to the local dialect, and then filled out the responses. Questionnaires were designed in the Department of Veterinary Public Health and Preventive Medicine, ABU Zaria and validated by pilot testing among 30 school-aged children in the area. The questionnaires were administered to the consenting students and information obtained was coded and analysed using SPSS version 17. Information sought included biodata, type of school they attend, area of residence, parents’ occupation, knowledge regarding rabies, source of information, and practice about the disease.

## 3. Results

Table 1 shows the biodata of the respondents. Among those receiving formal education, 55.8 % were boys while 37.5 % were girls. All 77 respondents (100%) of those receiving informal education (*almajiris*) were boys. Majority (75.5%) of the respondents reside outside ABU campus and for those in formal schools, 57.3%, 17.5% and 17.3% of their parents were civil servants, farmers and traders respectively.

**Table 1:** Demographics of children included in the study on knowledge and practice about rabies in Samaru, Zaria

Items	Children receiving formal Education (CRFE)	Children receiving informal education (CRIE)
**Gender**	**n_1_=400 (%)**	**n_2_=77(%)**
Male	223 (55.8)	77 (100)
Female	150 (37.5)	0 (0)
Not stated	27 (6.7)	0 (0)
Total	400 (100)	77 (100)
**Age**		
5-10	46 (11.5)	19 (24.8)
11-15	279 (69.8)	55 (71.4)
16-20	60 (15)	3 (3.9)
Not stated	15 (3.8)	0 (0)
Total	400 (100)	77 (100)
**Type of school**		
Formal	394 (98.5)	0 (0)
Informal	0 (0)	77 (100)
Not stated	6 (1.5)	0 (0)
total	400 (100)	77 (100)
**Residence**		
Inside ABU	90 (22.5)	0 (0)
Outside ABU	302 (75.5)	77 (100)
Not stated	8 (2)	0 (0)
Total	400 (100)	77 (100)
**Parents’ occupation**		
Civil servants	229 (57.3)	10 (13)
Farmers/	70 (17.5)	33 (42.9)
Traders	69 (17.3)	26 (33.8)
Not stated	33 (8.3)	8 (10.4)
Total	400 (100)	77 (100)
***χ^2^=53.75, df=3, p=0.0000***		

Number in front and percentages in parentheses

Table 2 shows the respondents’ knowledge and source of information about rabies. Here, 50.8% of those in the formal schools indicated they knew what rabies is while in the informal educational setting, only 32.5% said they knew about rabies. Those in the formal schools obtained information mainly from friends and at school (25.9% each), on television (TV) (21.1%), radio (10.3%) and 12.3% from other means while 4.4% said that they could not remember how they learnt about the disease. In the informal setting, however, the major source of information was from friends (56%), followed by from school (16%) and other means (16%). Only 4% knew about rabies from the radio while none (0%) indicated obtaining rabies knowledge from TV.

**Table 2:** Knowledge regarding rabies among children receiving formal and informal education in Samaru, Zaria, Nigeria

Items	Formal education n_1_ (%)	Informal education n_2_ (%)
**Awareness of rabies (n_1_=400, n_2_=77)**		
Yes	203 (50.8)	25 (32.5)
No	184 (46)	47 (61)
Not stated	13 (3.3)	5 (6.4)
total	400 (100)	77 (100)
**Source of Information (n_1_=203, n_2_=25)**		
School	53 (25.9)	4 (16)
Friends	53 (25.9)	14 (56)
T.V	43 (21.1)	0 (0)
Radio	21 (10.3)	1 94)
Others	25 (12.3)	4 (16)
Dont remember	8 (4.4)	2 (8)
Total	203 (100)	25 (100)
**Mode of Transmission (n_1_=203, n_2_=25)**		
Dog bite	127 (65.7)	2 (8)
I don’t know	39 (19.2)	18 (72)
Others	11 (5.4)	5 (20)
Not stated	26 (12.8)	0 (0)
Total	203 (100)	25 (100)
**Prevention (n_1_=203, n_2_=25)**		
Yes	179 (86.5)	21 (84)
No	24 (13.5)	13 (52)
Not stated	0 (0)	1 (4)
Total	203 9100)	25 (100)
**Signs of rabies (n_1_=203, n_2_=25)**		
Aggression	53 (26.2)	3 (12)
Paralysis	23 (11.2)	2 (8)
Salivation	50 (24.3)	3 (12)
Biting	62 (30.2)	10 (40)
Multiple options	20 (9.7)	7 (28)
Total	202 (100)	25 (100)

Number in front and percentages in parentheses

**Table 3:** Practice regarding rabies among children receiving formal and informal education in Samaru

Items	Formal education n_1_ (%)	Informal education n_2_ (%)
**If they own a dog (n_1_=400, n_2_=77)**		
Yes	106 (26.6)	9 (11.7)
No	293 (73.4)	65 (84.4)
Not stated	1 (0.3)	3 (3.9)
total	400 (100)	77 (100)
**Vaccination of dogs (n_1_=106, n_2_=9)**		
Yes	80 (75.5)	1 (11.1)
No	22 (20.8)	65 (84.4)
Not stated	4 (3.8)	69 913)
Total	106 9100)	9 (100)
**If they play with dogs (n_1_=400, n_2_=77)**		
Dogs i know	93 (23.3)	11 (14.3)
Any dog i like	38 (9.5)	8 (10.4)
I don’t play with dogs	237 (59.3)	46 959.7)
Not stated	32 (8)	12 (15.6)
Total	400 (100)	77 (100)
***χ^2^=6.46, df=3, p=0.09***		
**If bitten by a dog (n_1_=400, n_2_=77)**		
Yes	102 (25.5)	8 (10.4)
No	285 (71.3)	63 (81.8)
Not stated	13 (3.3)	6 (7.8)
Total	400 (100)	77 (100)
**Site of bite (n_1_=102, n_2_=8)**		
Head	7 (6)	1 (12.5)
Hands	20 (17.1)	5 (62.5)
Leg	42 (35.9)	1 (12.5)
Back	7 (6)	0 (0)
Others	41 (35)	0 (0)
Not stated	0 (0)	1 (12.5)
Total	117 (100)	8 9100)
**Circumstance of bite (n_1_=102, n_2_=8)**		
Provoked	38 (37.3)	5 (62.5)
Unprovoked	29 (28.4)	3 (37.5)
Not stated	35 (34.3)	0 (0)
Total	102 (100)	8 (100)
**Reported to (n_1_=102, n_2_=8)**		
Parent	53 (52)	0 (0)
Teacher	22 (21.6)	7 (87.5)
Both	35 (34.3)	1 (12.5)
Not stated	2 (1.9)	0 (0)
Total	102 (100)	8 (100)
**What happened to dog(n_1_=102, n_2_=8)**		
Killed	7 (6.9)	1 (12.5)
Ran away	20 (19.6)	1 (12.5)
Taken to vet	28 (27.7)	0 (0)
Not stated	47 (46)	6 (75)
Total	102 (100)	8 (100)
**Bite treatment (n_1_=102, n_2_=9)**		
Traditional	13 (12.7)	1 (12.5)
Hospital	65 (63.7)	7 (87.5)
None	2 (1.9)	0 (0)
Others	6 (5.9)	0 (0)
Not stated	16 (15.7)	0 (0)
Total	102 (100)	8 (100)

Number in front and percentages in parentheses

Regarding the mode of transmission, only 2.5% in the informal setting knew that dog bite could transmit rabies. Among children receiving formal education, only 32% knew that dog bite could transmit rabies. When asked if they knew that rabies could be prevented, among those who said they knew about rabies, 86.5% of those in the formal school setting and 84% of those in the informal school setting answered yes. When asked questions to see if they could recognise rabid dogs, 56.6% and 52% of children in formal and informal settings respectively matched rabies with aggressive behaviour and biting while only 11.2% and 8% of the respective categories associated rabies with paralysis.

Regarding practices, 26.6% of families of children receiving formal education owned dogs while only 11.7% of families of children in the informal educational setting kept dogs. When asked if their dogs were vaccinated against rabies, 75.5% of the children in formal schools answered yes while only 11.1% answered in the affirmative among children in informal schools. From our results, 23.3% of children receiving formal education said they play with dogs they know and another 9.5% said they play with any dog they like. Among children receiving informal education, however, 14.3% and 10.4% answered yes to the respective questions. Furthermore, 25.5% and 10.4% of children in formal and informal schools respectively said they had a previous history of dog bite. Among these children, 37.3% and 62.5% of bite circumstances were due to provocation as reported by children in formal and informal schools respectively.

Among children bitten by dogs, 52% and 0% in the formal and informal settings respectively reported to parents, while 21.6% and 87.5% reported to teachers in the two settings. In many cases, the offending dogs were killed (26.5%) or they got away (25%) as reported by children in the formal and informal school settings, while 27.5% and 0% of the children in the formal and informal schools responded that they reported dog bites to veterinary clinics. From responses gathered, 63.7% and 87.5% of the children bitten by dogs in the formal and informal schools respectively received hospital treatment while the others received other forms of treatment such as African traditional treatment, prayers etc.

## 4. Discussions

There was an association between parents’ occupation and type of school the children were attending (x^2^=53.75, p<0.05). Up to 54% of the children attending formal schools came from homes where the parents were civil servants while 43% of those attending informal schools said their parents were farmers. This is not suprising to see since civil servants understand the importance of formal schools and it is expected that they should enrol their children in formal schools.

More children receiving formal education (50.8%) than those receiving informal education (32.5%) knew about rabies. In the past, rabies awareness lectures have been organised by the Faculty of Veterinary Medicine, Ahmadu Bello University, Zaria as part of the World Rabies Day events (www.worldrabiesday.org), reaching some formal schools in the area (Dzikwi et al., 2011a). This might have contributed to the knowledge about rabies among children in this category of respondents. Regarding source of information about rabies, children in the informal setting learn more from talking with their friends ie information is passed best from friend to friend, orally. This method of transfer of information is commonly utilised in the traditional African setting and is not surprising to see children in the informal setting transferring information by word of mouth. It is however necessary for information to be accurate for it to be usefull. It will be meaningful then to educate them well so that they can share accurate information about the disease their other friends. Children in the formal schools have access to mass media. However, for the *almajiris*, the mass media (radio and TV) may not be a very useful source of information because of the special circumstances under which they are trained which limits their access to electronic media either for information or for recreation.

Regarding knowledge of means of transmission of the disease, 32% and 2.9% of children in the formal and informal schools knew how rabies is transmitted. The remaining either did not know, or stated incorrect means such as walking bare-footed, or rabies being food-borne etc. This is very alarming and unacceptable since they may completely ignore dog bites because they are unaware of the potential hazards. For rabies prevention to be achieved, children should be able to recognise possible exposures and the implications so that they can report promptly. The paralytic form of rabies appears to be unrecognised by both categories of children and this could have implications since exposures may therefore not be recognised.

Vaccination of dogs against rabies is important for ultimately controlling the disease but many dog owners do not vaccinate their animals. Even though up to 76% of children in the formal setting said their dogs were vaccinated, this may not be the true situation based on previous study in this locality where only about 17% said they vaccinated their dogs (Dzikwi et al., 2011b). Furthermore, it is not easy to verify this claim since vaccinated dogs are not tagged. It is also possible that the dogs have received veterinary attention for some other reason in the past, which the respondents interpreted as rabies vaccination. Responsible dog ownership is mandatory for rabies control. Even though laws exist in Nigeria which mandate vaccination and proper identification as well as keeping dogs on leash among other things, these are however not enforced and are partly responsible for our current endemic rabies status.

Many children in the study said they played with dogs among which include dogs they do not know. There was however no association between playing with dogs and type of schools the children were attending (x2=6.46, p>0.05). Children are known to play and even out-rightly provoke dogs by throwing stones and other objects at them. This practice is risky since dogs could react by biting. Hence, children should be discouraged from provoking dogs.

Majority of the children in formal schools who have been bitten by a dog reported to their parents (52%) and 21.6% to their teachers, while amongst those in informal schools, they reported only to their teachers (87.5%). Since *almajiris* are under the care of their teachers, it is important that these malams be properly educated about appropriate first aid and follow up when any case of dog bite is reported to them. Parents and school teachers also need to be properly educated to ensure they give the right care to their children and wards. If a parent or guardian knew a child was exposed to a rabid dog, knowing the implication, they would seek post-exposure prophylaxis, but children who do not report dog bites to an adult are most often the victims of rabies (Briggs & Mahendra, 2007).

Only 27.7% respondents said the biting dogs were taken to veterinary hospitals. It is important to educate people on the need to institute first aid by washing the bite wound with soap and water and then report with the offending dogs to a veterinary clinic for the dogs to be assessed and quarantined if deemed necessary by a veterinarian. This practice also ensures that the veterinarians and medical personnel work together since the final diagnosis in the animal is needed for full and appropriate treatment of the bite victims. Many people still rely on traditional African medicine as a form of treatment for dog bite. Unfortunately, these are ultimately not useful in rabies prevention. In a report from India, 100% (8/8) of the patients who resorted to indigenous methods of treatment following exposure to rabies by dog-bite died even though they later sought hospital treatment, it was too late (Dutta, 2002). Appropriate and prompt post-exposure treatment is highly recommended especially where stray dogs are involved because about 10% of these dogs are rabid (Wilde et al., 1991). Where the children said they received hospital treatment, many probably went to nearby chemists as is the common practice among many people in this region. If this is the case then probably they did not receive antirabies biological ie vaccines and rabies immunoglobulin because they are not readily available and where they are available, they are expensive.

## 5. Conclusions

The children’s level of awareness about rabies is poor. Majority do not know about the paralytic form of rabies and some do not know how the disease is transmitted hence they may overlook potential exposures. The practise regarding dogs and rabies such as vaccination of dogs is not adequate. Furthermore, appropriate post-exposure treatment is not sought and the mass media are not exploited for rabies education.

## 6. Recommendations

Accurate rabies education with emphasis on transmission and potential exposure as well as first aid treatment should target children regardless of the type of education they are receiving. Possible inclusion of rabies education in health science subjects in schools could be useful. We also recommend rabies education for parents and school teachers in both the formal and informal setting so that they can take appropriate and prompt actions like immediate washing of bite wounds and reporting to the nearest hospital, where dog bites have occurred. Enforcement of the laws regarding dog ownership is also vital to control rabies in Nigeria.

## 7. Strenghts and Limitations

The strengths of the study is that it provides baseline information in a new area which will be used to design meaningful and appropriate intervention strategies for rabies control in this locality. The limitations of the study are the inability to verify some claims by the respondents and the study had to rely solely on the responses provided.
